# Fossil and Genetic Evidence for the Polyphyletic Nature of the Planktonic Foraminifera "*Globigerinoides*", and Description of the New Genus *Trilobatus*

**DOI:** 10.1371/journal.pone.0128108

**Published:** 2015-05-28

**Authors:** Silvia Spezzaferri, Michal Kucera, Paul Nicholas Pearson, Bridget Susan Wade, Sacha Rappo, Christopher Robert Poole, Raphaël Morard, Claudio Stalder

**Affiliations:** 1 Department of Geosciences, University of Fribourg, Fribourg, Switzerland; 2 Center for Marine Environmental Sciences MARUM, University of Bremen, Bremen, Germany; 3 School of Earth & Ocean Sciences, Cardiff University, Cardiff, United Kingdom; 4 Department of Earth Sciences, University College London, London, United Kingdom; Ben Gurion University of the Negev, ISRAEL

## Abstract

Planktonic foraminifera are one of the most abundant and diverse protists in the oceans. Their utility as paleo proxies requires rigorous taxonomy and comparison with living and genetically related counterparts. We merge genetic and fossil evidence of “*Globigerinoides*”, characterized by supplementary apertures on spiral side, in a new approach to trace their “total evidence phylogeny” since their first appearance in the latest Paleogene. Combined fossil and molecular genetic data indicate that this genus, as traditionally understood, is polyphyletic. Both datasets indicate the existence of two distinct lineages that evolved independently. One group includes “*Globigerinoides*” *trilobus* and its descendants, the extant *“Globigerinoides*” *sacculifer*, *Orbulina universa* and *Sphaeroidinella dehiscens*. The second group includes the *Globigerinoides ruber* clade with the extant *G*. *conglobatus* and *G*. *elongatus* and ancestors. In molecular phylogenies, the *trilobus* group is not the sister taxon of the *ruber* group. The *ruber* group clusters consistently together with the modern *Globoturborotalita rubescens* as a sister taxon. The re-analysis of the fossil record indicates that the first “*Globigerinoides*” in the late Oligocene are ancestral to the *trilobus* group, whereas the *ruber* group first appeared at the base of the Miocene with representatives distinct from the *trilobus* group. Therefore, polyphyly of the genus "*Globigerinoides*" as currently defined can only be avoided either by broadening the genus concept to include *G*. *rubescens* and a large number of fossil species without supplementary apertures, or if the *trilobus* group is assigned to a separate genus. Since the former is not feasible due to the lack of a clear diagnosis for such a broad genus, we erect a new genus *Trilobatus* for the *trilobus* group (type species *Globigerina triloba* Reuss) and amend *Globoturborotalita* and *Globigerinoides* to clarify morphology and wall textures of these genera. In the new concept, *Trilobatus* n. gen. is paraphyletic and gave rise to the *Praeorbulina* / *Orbulina* and *Sphaeroidinellopsis* / *Sphaeroidinella* lineages.

## Introduction

Foraminifera are eukaryotic unicellular protists with a biomineralized shell representing one of the most diverse groups in the modern oceans [1]. About 20% of the estimated 5.8 billion tonnes of carbonate produced annually is composed of foraminiferal shells, which constitute around 70% of the sediments on ocean floors [2]. Planktonic foraminifera can range, numerically, from 1.9 to 9.9% of the total zooplankton abundance, locally reaching dominance [3]. Their highest standing stock concentration (over 10^4^ specimens per 1000m^3^) has been recorded in major current systems, boundary currents, divergence and upwelling regions [4]. As a major constituent of microzooplankton, they are key components of marine foodwebs and the main predator of phytoplankton in tropical and subtropical oligotrophic waters. However, their role in the trophic chain is not completely understood [5].

Planktonic foraminifera have produced an exceptional fossil record, revealing an unparallelled archive of biodiversity, morphological and evolutionary change [6], and are commonly used as a proxy for paleoceanographic reconstructions. However, the use of planktonic foraminifera as paleo proxies implies and requires rigorous and consistent taxonomy, precise assessment of functional morphologies and their relation with autecology, biogeography, biodiversity and comparison with living and genetically related counterparts. In this research we merge genetic and fossil evidence in a new approach to “total evidence phylogeny” of the most abundant group of planktonic foraminifera, which have dominated the world’s oceans in temperate to tropical regions, since the Oligocene: the “*Globigerinoides*” (Note: for clarity we use quotation marks when referring to the classical concept of this genus).

“*Globigerinoides*” as classically understood (e.g., [7,8]) includes all Neogene planktonic foraminifera with globigeriniform morphology and supplementary apertures on the spiral side [9]. Representatives of the genus are extensively used to generate paleoceanographic and paleoclimatic reconstructions based on geochemical analyses of their shells. In addition, their diversity and abundance throughout the Neogene has been utilized for biostratigraphy and biochronology (e.g., [10,11,8,12,13]). “*Globigerinoides*" appears to have diversified in the earliest Miocene, with several intervals of radiation throughout the Miocene and Pliocene including the stratigraphically important development of *Praeorbulina-Orbulina* and *Sphaeroidinella* lineages (e.g., [14]).

The ancestry and early phylogeny of “*Globigerinoides*” is uncertain and has been debated for several decades. Although the idea that "*Globigerinoides*" is polyphyletic has been proposed many times by paleontologists (e.g., [7]), hitherto there has been little agreement about the actual number of separate origins and which morphospecies should be included in which group. For instance, Blow and Banner [15] proposed an evolutionary trend from *Globigerina praebulloides occlusa* to *Globigerinoides primordius*, which they considered the first representative of the genus. Takayanagi and Saito [16] identified two different groups of “*Globigerinoides*” based on the position of the primary aperture. One group shows the aperture placed on the sutures between the three last chambers (*Globigerinoides bollii*, *G*. *conglobatus*, *G*. *immaturus*, *G*. *obliquus*, *G*. *sacculifer* and *G*. *trilobus*) and the second group shows the aperture on the sutures between the penultimate and antepenultimate chambers (*G*. *elongatus*, *G*. *cyclostomus* and *G*. *ruber*). These pioneering studies on this group did not take into account wall textures but were based only on morphological features. Kennett and Srinivasan [7] identified one lineage of “*Globigerinoides*” originating from *Globigerina* sensu stricto with *bulloides*-type spinose wall texture typical of *Globigerina*, and one lineage evolving from *Zeaglobigerina woodi* with spinose and cancellate wall texture. They stated that their observation of several species of “*Globigerinoides*” evolving from different ancestors demonstrates that the genus is polyphyletic and therefore “artificial” (p. 51 in [7]). Keller [17] identified three lineages leading to a *Globigerinoides*-like morphology: one originating from *Globigerina praebulloides*, the second originating from *Globigerina woodi* and the third originating from *Globigerina connecta* (both species now assigned to the genus *Globoturborotalita*; see also fig 5 in [14]). Spezzaferri [12] also distinguished three groups of *Globigerinoides*, the first evolving from *Globigerina* with a *bulloides*-type wall texture of [18], and the second characterized by a cancellate, honeycomb wall texture and subdivided in two subgroups displaying the *ruber*- and *sacculifer*- type wall textures of [18]. A polyphyletic origin for the group was also postulated by [19] and [20]. The differences in wall texture in the two main groups may be associated with different ontogenetic development. An investigation of the ontogeny of two extant species in the group [21] revealed that in the juvenile stage, "*G*." *sacculifer* has subquadrate chambers forming a non-lobate test and pores present along both spiral and umbilical sutures whereas *G*. *ruber* has hemispherical chambers forming a lobate test and pores on the spiral side only.

Next to the type species *Globigerinoides ruber*, the genus also comprises “*Globigerinoides*” *trilobus* and “*G*.” *sacculifer* [22]. Note that modern *trilobus* and *sacculifer* are morphotypes (without and with a sac-shaped final chamber) of the same biological species [23,24], but the *trilobus* morphospecies appeared first in the fossil record so tends to be split by paleontologists (e.g., [14]).

Recent study and revision of the taxonomy of Oligocene and early Miocene planktonic foraminifera conducted by the Paleogene Planktonic Foraminiferal Working Group (PPFWG) has confirmed the long-standing view that modern “*Globigerinoides*” is polyphyletic. However, unlike in earlier investigations, there now exists an opportunity to independently test the validity of phylogenetic relationships in planktonic foraminifera hypothesized by the analysis of their fossil record. Since all main lineages of the genus “*Globigerinoides*”, as well as the hypothetical ancestral *Globoturborotalita* have extant representatives, the monophyly of “*Globigerinoides*” can be directly assessed by a molecular phylogenetic approach. In a joint effort between the PPFWG and the Scientific Committee on Oceanic Research/International Geosphere-Biosphere Programme (SCOR/IGBP) Working Group 138 “Planktonic foraminifera and ocean changes”, a detailed revision of the genus “*Globigerinoides*” has been carried out, combining fossil and molecular genetic evidence.

## Material and Methods

### Investigation of Fossil Samples

Ethics statement: The field collections carried out for the purpose of this paper did not involve endangered or protected species. No specific permission was required to collect the analyzed plankton. The sampling for fossil specimens was carried out in the open ocean and followed the regulations for the exclusive economic zones (EEZ) of the coastal countries, provided for each expedition by the respective authorities to the Deep Sea Drilling Project, Ocean Drilling Program and Integrated Ocean Drilling Program. These International Programs provided to the Authors the samples for this study. No permission was needed to collect samples from outcrops. No permits were required for the described study, which complied with all relevant regulations. The locations and other details on the investigated sites and of the new genus are in S1 Table.

Samples for the study of fossil “*Globigerinoides*” were prepared using standard techniques for Oligocene and Miocene foraminiferal investigation [12, 25]. Over a thousand samples from multiple ocean and outcrops sites were investigated—e.g., all the sites of [12]; the Caribbean and Trinidad sites of [26] (a duplicate of Bolli’s collection is stored in Fribourg), ODP Hole 1137A on the Kerguelen Plateau, the Aquitanian Global Boundary Stratotype Section and Point (GSSP) Lemme [27, 28], and the Martillac Section outcropping in the Aquitanian basin and adjacent to the historical stratotype of the Burdigalian [29]. Samples were weighed and then washed in distilled water. They were sieved into three size fractions >250 μm, 125–250 μm, and 40–125 μm, each fraction was weighed. The 40 μm mesh sieve was used to retain very small specimens and juveniles for eventual comparison.

The investigation of the early “*Globigerinoides*” was carried out in samples spanning the interval from the late Oligocene to the early Miocene, Zones O6 to M3. The zonal scheme of [13] was applied to place all samples in a consistent time frame. Species abundances were also investigated quantitatively in most of these samples [12, 25, 27]. The morphological and wall texture criteria that have been adopted to identify genera and species are based on comparison with SEM images of holotypes and on [22].

A morphometric study was conducted to assess the morphological affinity of the earliest “*Globigerinoides*”. To this end, samples DSDP Hole 538A-2CC (upper Oligocene, Zone O7, Gulf of Mexico), DSDP Hole 151-5-2, 98–99 cm (lower Miocene, Zone M1a, Gulf of Mexico) and Sample PJ262 (K3 F40-78) (lower Miocene, Trinidad, upper Zone M3) spanning the interval of appearance and diversification of “*Globigerinoides*” were chosen. Zone M3 has been identified by the co-occurrence of *Catapsydrax dissimilis* and *Globigerinatella* sp. Although [13, 30] have calibrated the last common occurrence of *“G*.*” primordius* at 24.3 Ma, this species is still present but very rare until Zone M3. At the beginning of their range the representatives of the genus “*Globigerinoides*” are generally not very abundant and present mainly at low latitudes, hence the choice of the samples in the Gulf of Mexico or at comparable paleolatitudes.

For morphometric analyses, the choice of specimens for this investigation is made following [31]. A standard amount of 10 cm^3^ of sediment for each sample was sieved though the 40-μm mesh and the residues were spread over a picking tray. The first 200 specimens belonging to the genus “*Globigerinoides*” were manually picked and identified at species level by comparison with the holotypes. The morphospecies included in this investigation are “*G*.” *trilobus*, “*G*.” *immaturus*, “*G*.” *praeimmaturus*, “*G*.” *subsacculifer*, *G*. *quadrilobatus*, *G*. *parawoodi*, *G*. *bollii*, and *G*. *subquadratus* (measurements are provided as on-line supplementary material). Morphometric parameters were measured on oriented specimens, with the KEYENCE VHX-600 digital microscope at a magnification of 200X. Digital images of the measured tests are available upon request. Measures are provided in S2, S3 and S4 Tables.

**Fig 1:**
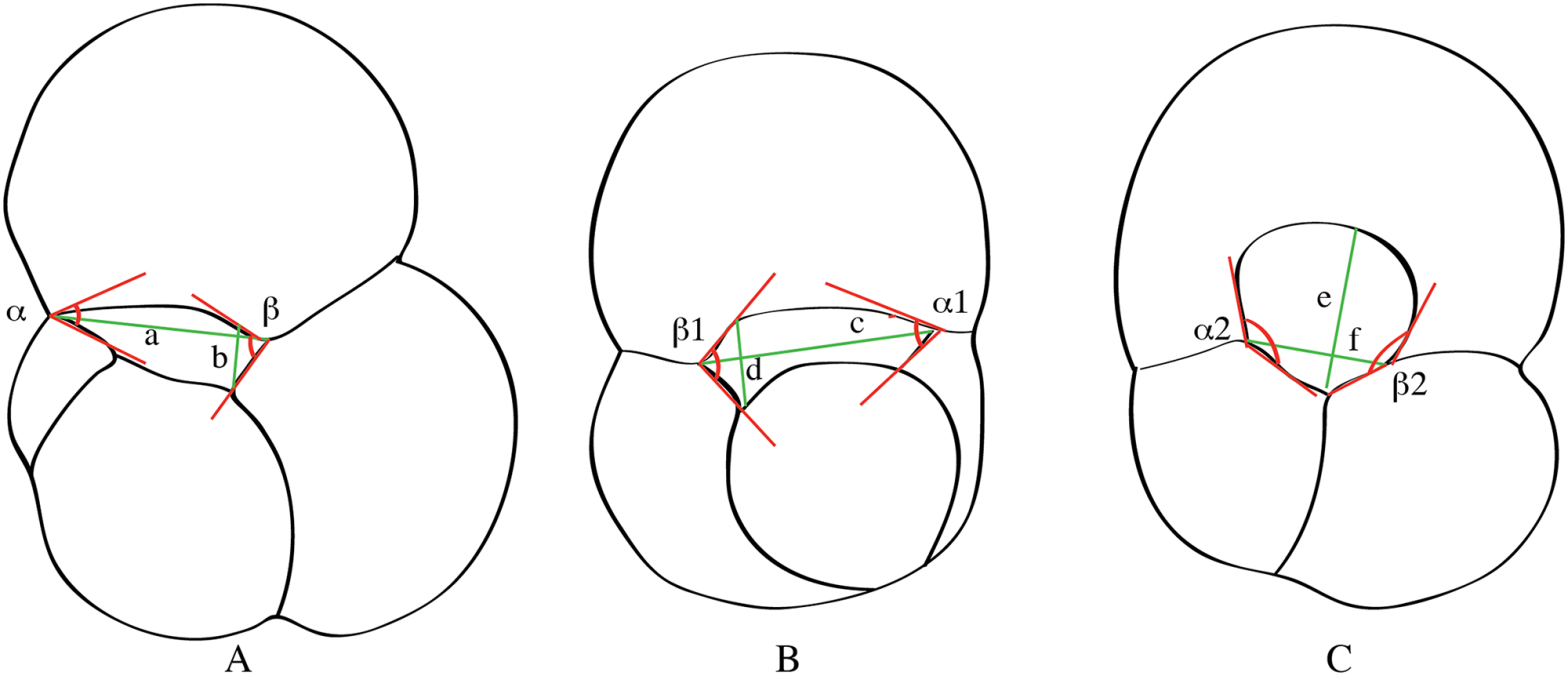
Definition of morphometric parameters used to evaluate the size and shape of the primary aperture in specimens with different morphologies (three- and four-chambered), exemplified for the morphospecies “*G*.” *primordius* (A), “*G*.”. *immaturus* (B), and *G*. *subquadratus* (C). Primary aperture diameter ratio (PADR) that describes the relative width of the aperture is defined as the ratio between aperture width and height (measured perpendicular to width), i.e. as a/b, c/d, f/e. The symmetry index (SI) indicates the degree of symmetry of the primary aperture and is expressed as the ratio between the larger and smaller angle at the opposite sides of the primary aperture, i.e. as β/α, β1/α1, β2/α2.

The morphometric parameters considered are the primary aperture diameter ratio (PADR, defined as the ratio between the maximum width of the aperture divided by the maximum height of the aperture measured from the position perpendicular to the maximum aperture width from the lower suture to the upper margin of the aperture) that indicates the height of the primary aperture and the symmetry index (SI = ratio between the larger angle divided by the smaller angle at the opposite sides of the primary aperture) that indicates the degree of symmetry of the primary aperture (Fig 1). The shape of the primary aperture is chosen as differentiating character because it has been identified to express the main difference among “*Globigerinoides*” in earlier studies (e.g., [26,16]).

### Molecular Phylogeny of “*Globigerinoides*”

To constrain the phylogenetic relationship within the extant members of the genus “*Globigerinoides*", a set of sequences representing a fragment of the small-subunit ribosomal DNA (SSU rDNA) in all extant species of planktonic foraminifera attributed to “*Globigerinoides*” as classically understood, its descendant (*Orbulina*), a presumed sister clade (*Sphaeroidinella*) and ancestral lineage (*Globoturborotalita*) were analysed. Sequences of the morphospecies *Globigerinoides ruber*, *Globigerinoides elongatus*, *Globigerinoides conglobatus*, “*Globigerinoides*” *sacculifer*, *Globoturborotalita rubescens* and *Orbulina universa* were downloaded from NCBI (National Center for Biotechnology information, http://www.ncbi.nlm.nih.gov/). Only sequences covering all six variable regions at the 3’ end of the SSU of foraminifera [32] were considered. For the phylogenetic analyses, we retained only one representative sequence of “*Globigerinoides*” *sacculifer*, *Globigerinoides conglobatus*, *Globoturborotalita rubescens* and *Orbulina universa*, two sequences representing two genetic types in *Globigerinoides ruber* (white), one sequence belonging to *Globigerinoides ruber* (pink) and two sequences representing two genetic types in *Globigerinoides elongatus* (Table 1). One sequence belonging to the species *Sphaeroidinella dehiscens* has been generated for the purpose of this study to replicate the results of [24], and confirm the identity of the sequences labelled as *S*. *dehiscens* recently deposited on NCBI (KJ633138, KJ633139, KJ633179-KJ633182). The obtained sequence was identical to those published but slightly longer, thus we retained it for further molecular investigation (see below). Inclusion of this species was deemed essential to ensure that the phylogenetic analysis in this study was comprehensive and did not suffer from artifacts due to incomplete taxon sampling.

**Table 1:** Details of SSU rDNA sequences used to generate the molecular phylogeny for extant *Globigerinoides*.

Accession	Included in	Morphospecies	Genetic type	Published by
Z83967	Ingroup	*Globigerinoides conglobatus*	NA	[38]
KM386666	Ingroup	*Sphaeroidinella dehiscens*	NA	This study
EU012458	Ingroup	*Globigerinoides ruber (white)*	Ia	[39]
EU012459	Ingroup	*Globigerinoides ruber (white)*	Ib2	[39]
Z83966	Ingroup	*Globigerinoides ruber (pink)*	NA	[38]
EU012452	Ingroup	*Globigerinoides elongatus*	IIa2	[39]
EU012463	Ingroup	*Globigerinoides elongatus*	IIa0	[39]
AB263459	Ingroup	*Globigerinoides sacculifer*	NA	[35]
JQ799894	Ingroup	*Globoturborotalita rubescens*	NA	[40]
AF102229	Ingroup	*Orbulina universa*	III	[38]
KF769946	Outgroup 1	*Beella digitata*	NA	[41]
Z83959	Outgroup 1	*Globigerinella siphonifera*	Ia	[38]
JQ743484	Outgroup 1	*Globigerinella siphonifera*	Ib	[40]
KF769861	Outgroup 1	*Globigerinella siphonifera*	IIIa	[41]
KF769820	Outgroup 1	*Globigerinella siphonifera*	IIb	[41]
KF769629	Outgroup 1	*Globigerinella siphonifera*	IIa2	[41]
KF769634	Outgroup 1	*Globigerinella siphonifera*	IIa3	[41]
KF769785	Outgroup 1	*Globigerinella siphonifera*	IIa5	[41]
Z83960	Outgroup 1	*Globigerinella siphonifera*	IIa3	[38]
U80788	Outgroup 1	*Globigerinella siphonifera*	IIa1	[42]
GQ293068	Outgroup 2	*Globigerina bulloides*	Ia	[43]
GQ293072	Outgroup 2	*Globigerina bulloides*	Ic	[43]
GU060421	Outgroup 2	*Globigerina bulloides*	Ie	[43]
GQ293070	Outgroup 2	*Globigerina bulloides*	Id	[43]
GQ293069	Outgroup 2	*Globigerina bulloides*	IIf	[43]
GQ293071	Outgroup 2	*Globigerina bulloides*	IIe	[43]
GU060422	Outgroup 2	*Globigerina bulloides*	IIa	[43]
AF250109	Outgroup 2	*Globigerina bulloides*	IIb	[44]
AY241713	Outgroup 2	*Globigerina bulloides*	IId	[45]
FJ643416	Outgroup 2	*Globigerina falconensis*	NA	[37]
AB263435	Outgroup 3	*Candeina nitida*	NA	[35]
AB263433	Outgroup 3	*Globigerinita glutinata*	NA	[35]
FJ643302	Outgroup 3	*Globigerinita uvula*	NA	[37]

Genetic types are isolated reproductive units with the exact same morphology. They form together what is traditionally recognized as a morphological species.

The new SSU rDNA sequence was obtained from a specimen of *Sphaeroidinella dehiscens* collected in the eastern tropical Pacific during the cruise SO226-3 of the RV SONNE on the 22^nd^ of March 2013 at station SO226/121 (15°176 N, 130.29 E) at water depth between 100 to 200 m using a multi-net with a mesh size of 100 μm [33]. The specimen was identified under a stereomicroscope, cleaned, photographed, transferred into 50 μl of GITC* (Guanidium Isothiocyanate) DNA extraction buffer, and stored at -20°C until DNA extraction was performed following the GITC* extraction procedure [34]. Amplification of a fragment of ~1000bp of the 3’ end of the SSU rDNA was conducted with the GoTaq polymerase (Promega) using the specific primer S14p (5'-AAGGGCACCACAAGMGCG-3’) [35] coupled with the universal primer 1528R (5'-TGATCCTTCTGCAGGTTCACCTAC-3’) [36] with an annealing temperature of 55°C. The amplified PCR (Polymerase Chain Reaction) product was purified using the QIAquick PCR purification kit (QIAGEN). The purified PCR product was sequenced directly by an external provider (LGC Genomics, Berlin). The sequence chromatogram was carefully checked and no multiple signal or sequencing artifact was observed. The resulting sequence has been deposited on NCBI under the accession number KM386666.

To assess the stability of the topology of the molecular phylogeny, the analysis was repeated with three different choices of outgroups. The outgroups were chosen to give an orientation of the evolution within the ingroup by locating the root at its basis, the ingroup being the members of the Genus *Globigerinoides*, *Orbulina* and *Globoturborotalita*. The outgroups consisted of a) representative sequences of the lineage *Globigerinella*, which in genetic phylogenies consistently appears as a sister lineage to “*Globigerinoides*” [37], b) representative sequences of *Globigerina*, which represents a less related lineage of spinose planktonic foraminifera and includes the highly derived sequence of *G*. *bulloides*, forming a long branch in molecular phylogenies [37] and c) three sequences of the phylogenetically distant microperforate clade, represented by the morphospecies *Globigerinita glutinata*, *Globigerinita uvula* and *Candeina nitida* (Table 1).

The alignment of the three sets of sequences was carried out using MAFFT v.7 [46] default options. This alignment algorithm was shown by [36] to yield the most consistent results. An experiment with multiple alignment strategies demonstrated that the topology of the phylogenetic tree in spinose planktonic foraminifera is largely robust to the choice of alignment algorithm. Best model of evolution was selected using jModeltest v. 2.1.4 [47]. The same model of evolution was retained for the three alignments (GTR+I+G). The most likely tree topology was inferred from the three alignments using a Maximum Likelihood Approach implemented in PhyML 3.0 software [48], using the selected model of evolution, NNI+SPR tree improvement and non-parametric bootstrapping (1000 pseudo replicates). The resulting trees were visualized with iTOL v 2.1 [49].

## Results

**Fig 2:**
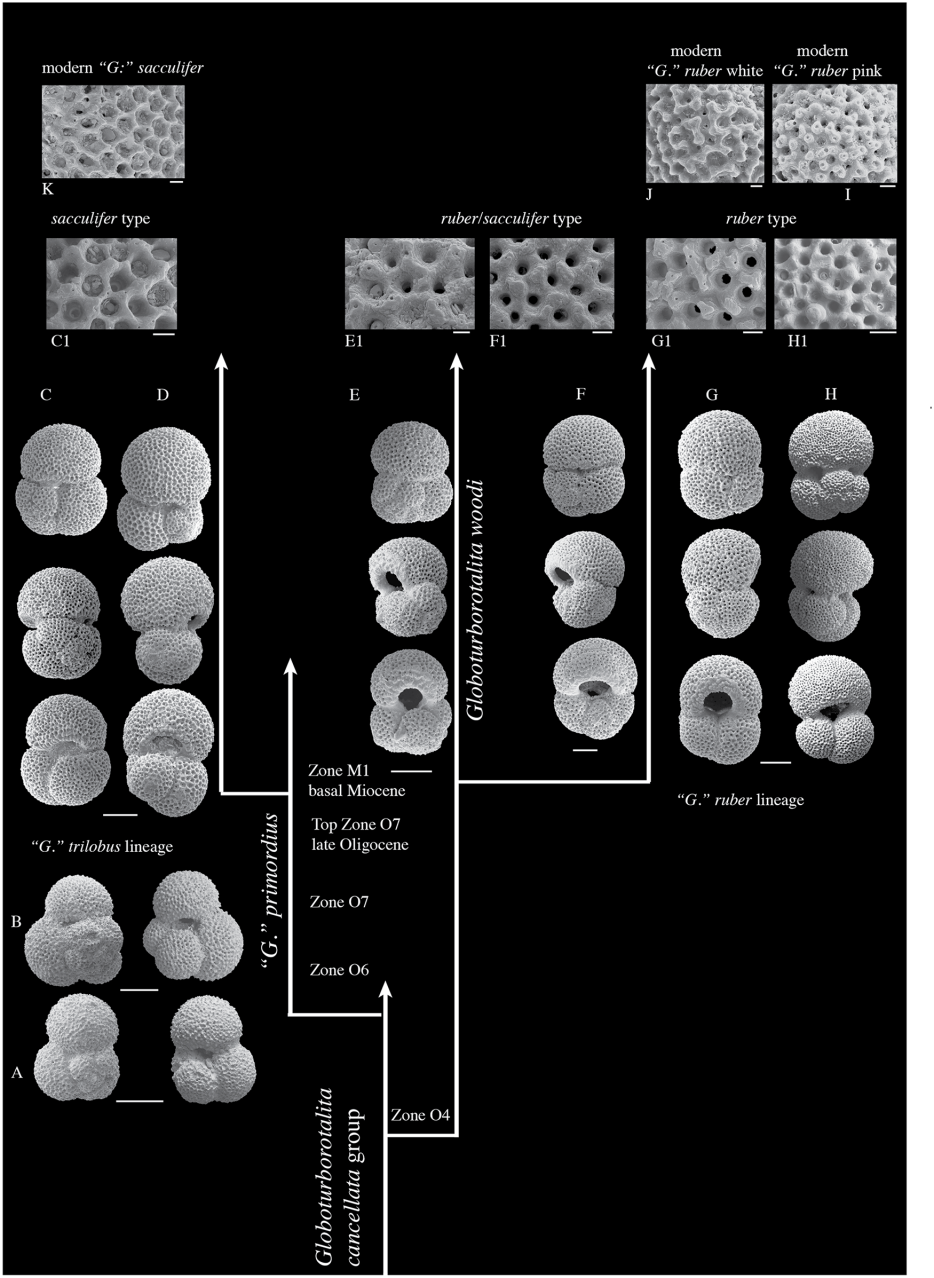
Chronology of the appearance of specimens with supplementary apertures on the spiral side showing the origin of the “*G*.” *trilobus* and *G*. *ruber* lineages from different *Globoturborotalita* ancestors at the Oligocene-Miocene transition. The “*G*.” *trilobus* lineage starts in the late Oligocene with the ancestor “*G*.” *primordius* and diversifies in the lower Miocene at the base of Zone M1. The *G*. *ruber* lineage starts at the base of Zone M1. A = “*Globigerinoides*” *primordius*, Sample K3-F10-76, Trinidad; B = “*Globigerinoides*” *primordius*, Sample DSDP Hole 538A-2CC, Gulf of Mexico; C = “*G*.” *praeimmaturus*, DSDP Hole 94-10-2, 22–24 cm, Gulf of Mexico; D = early form of “*G*.” *trilobus*, Sample Bolli 407, Trinidad; E = *Globoturborotalita woodi*, Sample DSDP Hole 94-10-2, 22–24 cm, Gulf of Mexico; F = *Globigerinoides parawoodi*, Sample DSDP Hole 94-10-2, 22–24 cm, Gulf of Mexico; G = *Globigerinoides subquadratus*, Sample DSDP Hole 94-10-2, 22–24 cm, Gulf of Mexico; H = *Globigerinoides* sp. 1, Sample Bolli 407, Trinidad; I = wall texture of modern *G*. *ruber* pink, Sample boxcore top BC3441, Alboran Sea; J = wall texture of modern *G*. *ruber* white, Sample boxcore top BC3441, Alboran Sea; K = wall texture of modern “*G*.” *sacculifer*, Sample boxcore top BC3441, Alboran Sea. Scale bars of all specimens = 100 μm; Scale bars of all wall textures = 10 μm. Zonation from [13].

The detailed re-investigation of the fossil record allowed us to trace the appearance of specimens with supplementary apertures on the spiral side. These first occurred in the late Oligocene (Zone O6-O7) at ~26 Ma. The chronology of the appearance of these specimens indicates two branches, with one leading to the “*G*.” *trilobus* lineage and a second leading to the *G*. *ruber* lineage (Fig 2). The first appearance of specimens with one or two supplementary apertures on the spiral side (“*G*.” *primordius*) involved forms with a strongly cancellate wall texture (*sacculifer*-type of [22]), and with a variety of morphologies, from elongated to subcircular tests, occurring in the late Oligocene Zone O6.

In the earliest Miocene (base of Zone M1a, within the total range distribution of *Paragloborotalia kugleri*) “*G*.” *primordius* evolved lower arched apertures and a more elongated and less lobate test tending to the typical morphology of “*G*.” *trilobus*. At the same level a new and distinct type of specimen with supplementary apertures on the spiral side appears. This type is associated with wider, more rounded and high arched primary apertures, a less well developed cancellate wall texture (*ruber/sacculifer*-type, [50]) and a lobate profile of the test tending to the typical morphology of *G*. *ruber* (Fig 2).

**Fig 3:**
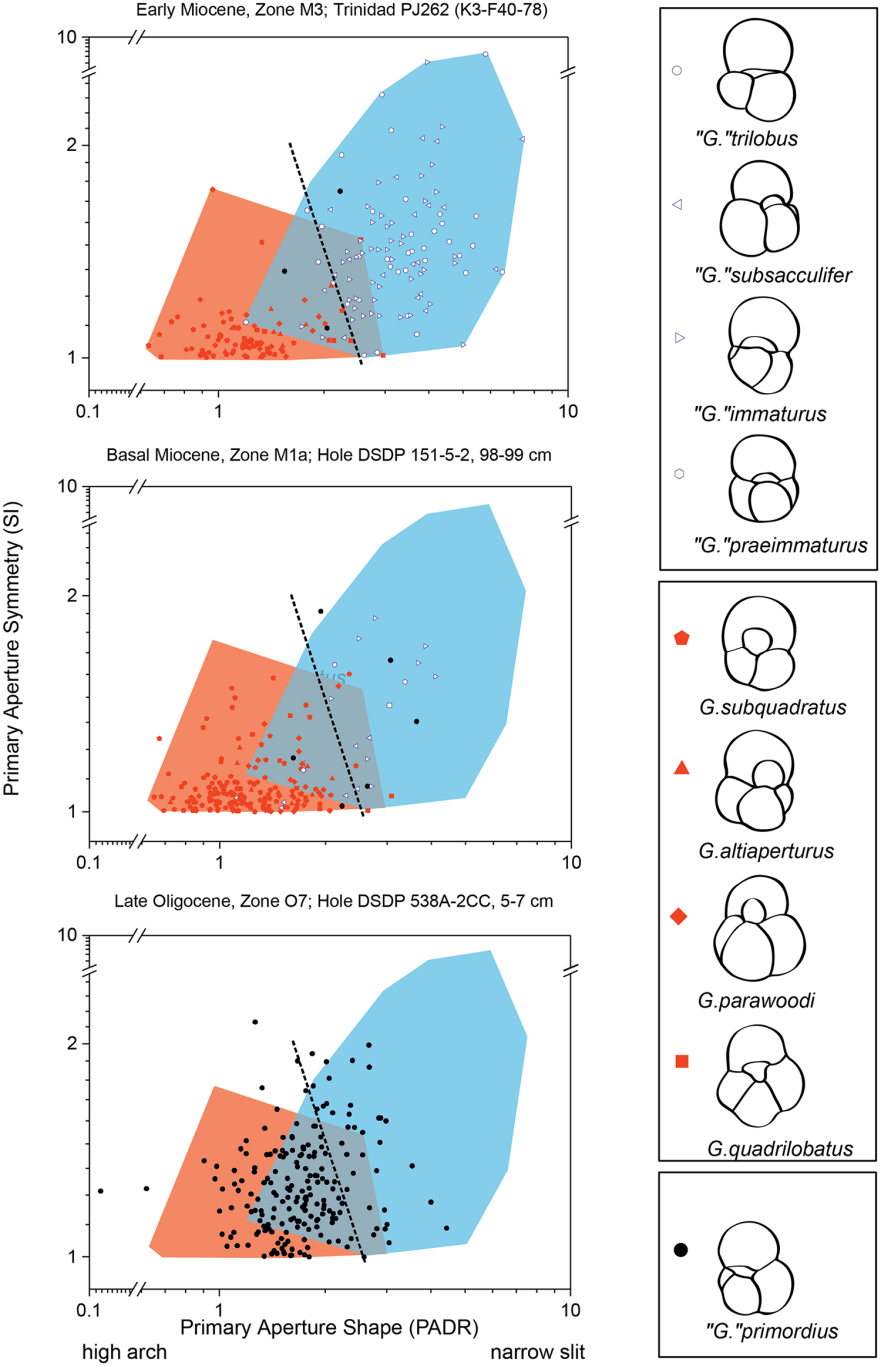
A morphometric analysis of the primary aperture among different morphologies of “*Globigerinoides*”. The Symmetry Index (SI) and the Primary Aperture Diameter Ratio (PADR) are defined in Fig 1. Dashed line indicates the best discrimination between specimens attributed to the *trilobus* (blue) and *ruber* (red) lineages, determined by linear discriminant analysis between the two groups in Zone M3. The line is perpendicular to the linear discriminant function at the position of the optimum discrimination score (z = 0). Specimens on each side of the line would be classified as belonging to either one of the groups in Zone M3.

The morphometric investigation on the aperture shape and position (PADR and SI) for three time slices from the late Oligocene to the early Miocene shows a clear distinction among these lineages, previously attributed to “*Globigerinoides*”. Most importantly, the analysis shows that populations of the late Oligocene morphospecies with supplementary apertures on the spiral side (“*G*.” *primordius*, Fig 3, DSDP Hole 538A) mostly occupy the part of the morphospace of the early Miocene “*G*. *trilobus*” lineage, whereas, specimens with low values of both parameters (PADR and SI), typical of the Miocene representatives of the *G*. *ruber* lineage are rarer in that morphospace (Fig 3, DSDP Hole 151-5-2, 98–99 cm and Sample K3 F10-76). Although some specimens of *“G*.*” primordius* may show some affinity to the *G*. *ruber* lineage, we consider its morphology as more consistent with being an ancestor of the *“G*.*” trilobus* lineage, which thus could have been derived morphologically from within the range of morphological variability already present in *“G*.*” primordius*.

**Fig 4:**
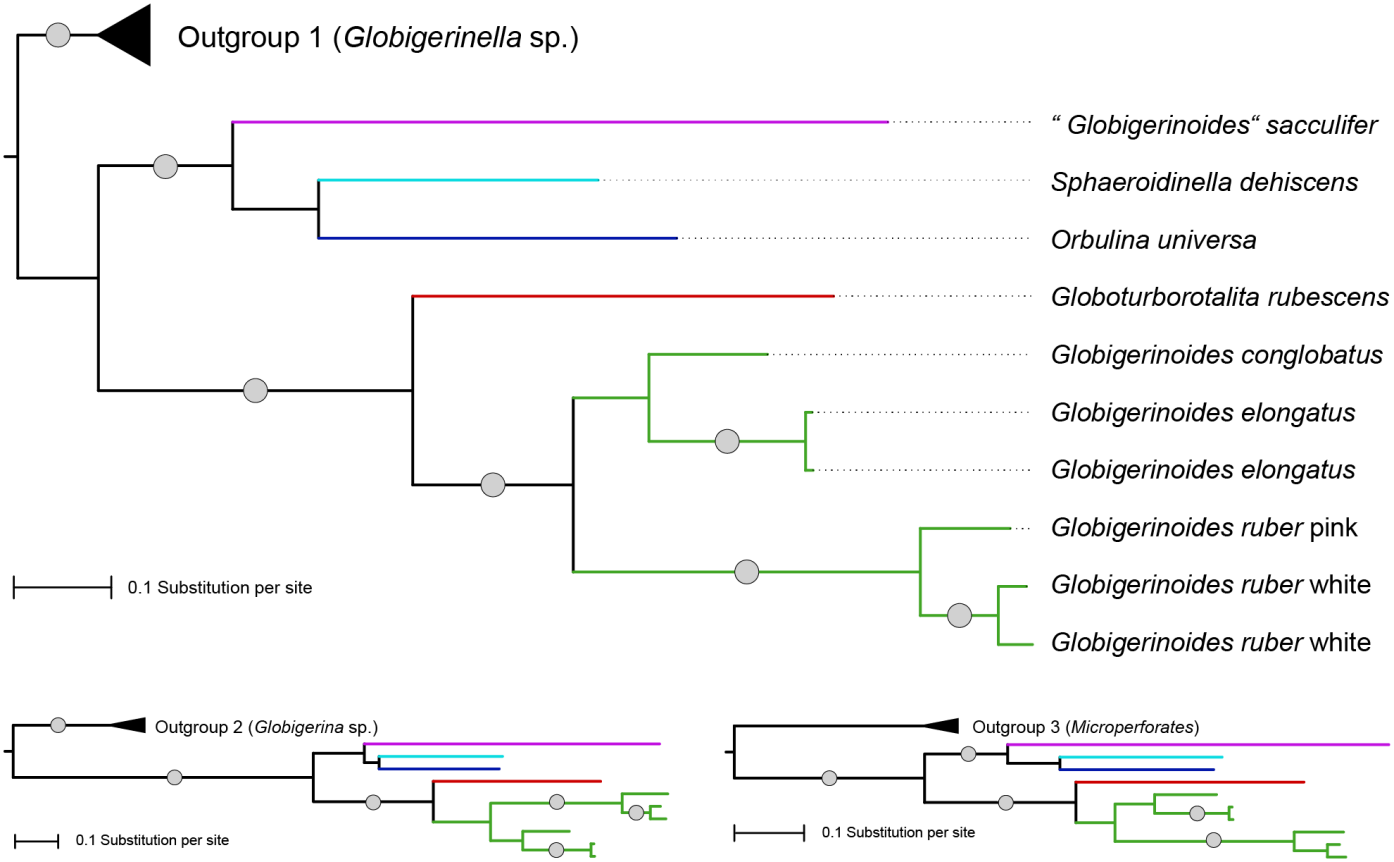
Maximum likelihood phylogeny of representative SSU rDNA sequences of extant species of the genus “*Globigerinoides*” and related taxa. The lower panels show the topology for trees rooted on different outgroups (Table 1). Branches with bootstrap support > 95% (1000 replicates) are marked with grey circles.

The existence of two distinct lineages within “*Globigerinoides*” implied by our analysis of the fossil record is corroborated by genetic data. Irrespective to the choice of outgroup, the phylogenetic analysis of SSU rDNA sequences indicates that the analysed sequences of the living representatives of the genus “*Globigerinoides*” belong to two lineages (Fig 4). In all analyses, the resulting topology is identical, suggesting that sequences of *Globigerinoides ruber*, *G*. *elongatus* and *G*. *conglobatus* form a clade together with *Globoturborotalita rubescens*, with bootstrap support > 95% in all three phylogenies. In contrast, the sequence of “*Globigerinoides*” *sacculifer* clusters with *Orbulina* and *Sphaeroidinella*, with bootstrap support > 95% in two of the three phylogenies. The branching order within this group remains unresolved because the depicted sister relationship between *Orbulina* and *Sphaeroidinella* is not supported by the bootstrap analysis. This means that the alternative topology, with “*Globigerinoides*” *sacculifer* and *Orbulina* forming a clade, which is sister to *Sphaeroidinella*, as suggested by the fossil record [14], cannot be rejected.

## Discussion

Although polyphyletic form-genera were frequently used for planktonic foraminifera in the past, modern taxonomic practice (e.g., p. 18 in [51]) is that all higher taxa must be monophyletic or paraphyletic. The SSU rDNA phylogeny provides strong support for a polyphyletic "*Globigerinoides*". The results are robust to the choice of outgroup and the high bootstrap values and comprehensive taxon sampling make any other interpretation of the molecular phylogeny highly unlikely. Given that phylogeny, the genus could be considered paraphyletic only if its concept were broadened to include *Globoturborotalita rubescens* and its ancestor and all their descendants, or if a different genus name was used for the “*G*.” *trilobus* group. In both cases, the thus amended genus *Globigerinoides* would require a new diagnosis, relying on characters other than simply the globigeriniform morphology and presence of supplementary apertures on the spiral side. Because *Globoturborotalita* does not possess supplementary apertures (e.g., [7]), this character must have evolved at least two times.

**Fig 5:**
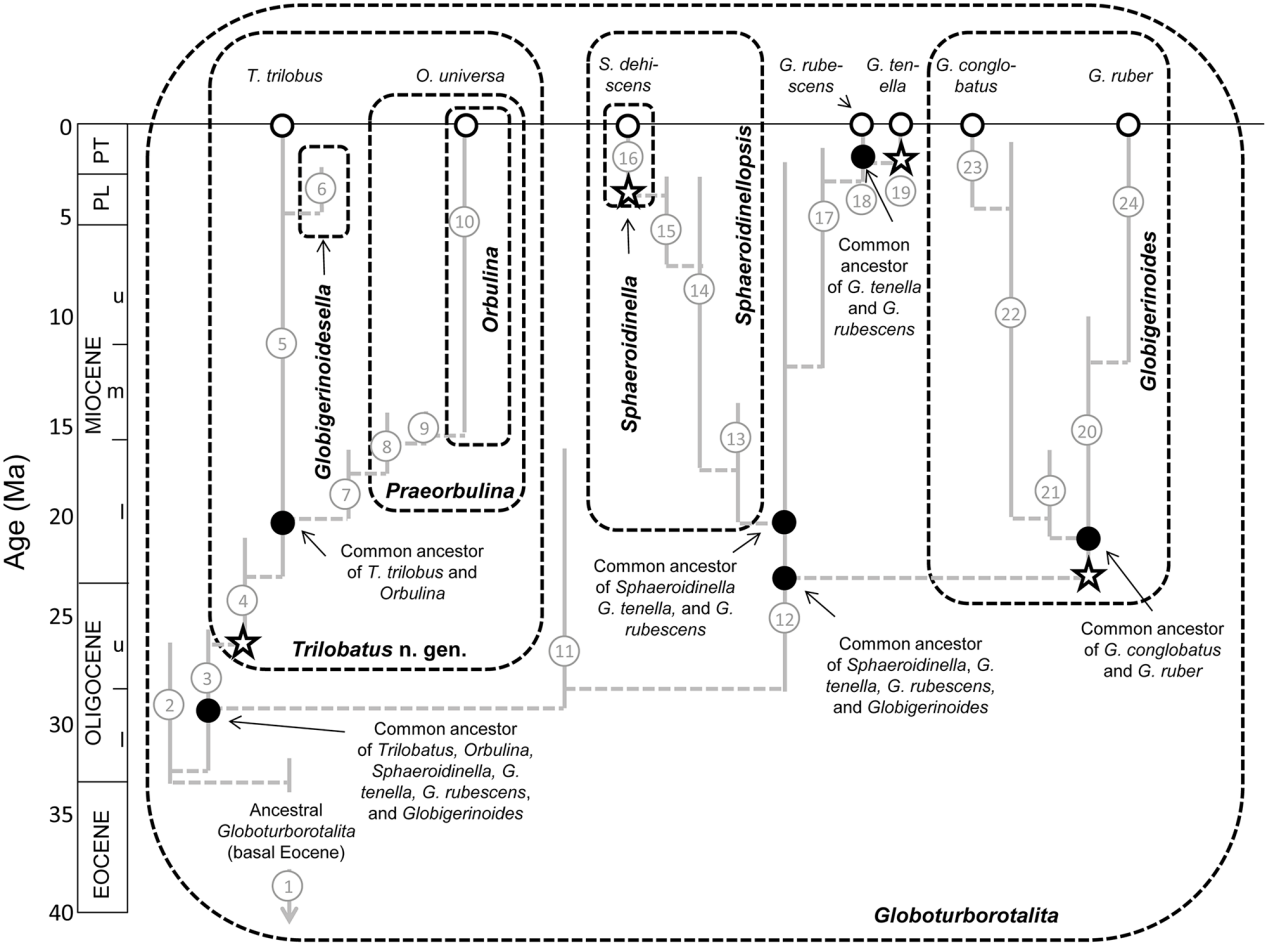
Simplified stratophenetic phylogeny based solely on fossil data (and thus not necessarily congruent with DNA-based phylogenies) showing the relationships between living species of *Sphaeroidinella*, *Trilobatus* n. gen., *Orbulina*, *Globigerinoides*, and *Globoturborotalita* (open circles) and their common ancestors (filled circles). Stratigraphic ranges are shown as vertical grey bars and evolutionary relationships by horizontal dashed grey lines. The reconstruction is based on the new observations of the PPFWG for the Eocene to lower Miocene and [14] for the rest. This is not the complete clade: many fossil species (side-branches that are not ancestral to the modern species) in the genera *Globoturborotalita*, *Trilobatus*, and *Globigerinoides* have been omitted for clarity. The modern species *Globigerinoides elongatus* is omitted because most paleontologists lumped it (wrongly) with *G*. *ruber* prior to genetic studies. Also omitted is the modern *Globigerinoides tenellus* which has often been considered as closely related with *G*. *rubescens* but that relationship is uncertain. The modern species *Trilobatus trilobus* encompasses *T*. *sacculifer* which has a shorter stratigraphic range, are shown. Genera are shown as dashed panels; paraphyletic genera are shown encompassing their descendant genera. The evolution of supplementary apertures is shown as stars; this occurred independently three times in the origin of *Trilobatus*, *Globigerinoides*, and *Sphaeroidinella*. Fossil morphospecies are given as numbered circles: (1) *Globoturborotalita bassriverensis*; (2, 3) *Globoturborotalita cancellata* group; (4) *Trilobatus primordius*; (5) *Trilobatus trilobus*; (6) *Trilobatus sacculifer*; (7) *Globigerinoidesella fistulosa*; (8) *Trilobatus bisphericus*; (9) *Trilobatus sicanus*; (10) *Praeorbulina glomerosa*; (11) *Orbulina universa*; (12) *Globoturborotalita brazieri*; (13) *Globoturborotalita woodi*; (14) *Sphaeroidinellopsis disjunctus*; (15) *Sphaeroidinellopsis seminulinus*; (16) *Sphaeroidinellopsis paenedehiscens*; (17) *Sphaeroidinella dehiscens*; (18) *Globoturborotalita decoraperta*; (19) *Globoturborotalita rubescens*; (20) *Globigerinoides subquadratus*; (21) *Globigerinoides altiaperturus*; (22) *Globigerinoides obliquus* / *extremus*; (23) *Globigerinoides conglobatus*; (24) *Globigerinoides ruber*. PL = Pliocene, PT = Pleistocene, e = early, m = middle, l = late. Timescale of [13] and [30].

The morphometric analysis of fossil populations shows that the genus “*Globigerinoides*” as currently understood includes two groups. The first group appeared in the late Oligocene and is characterized by variable size and shape of the aperture (Fig 3), as indicated by the wide range of PADR values. This group can be attributed to “*G*.” *primordius*. It presence persisted in through the early Miocene maintaining the same variability in the size and shape of the aperture (high values of PADR and SI). The second group is characterized by more uniform size and shape of the aperture, and in particular, the values of SI constantly between 1 and 1.5 indicate a generally symmetrical primary aperture, characteristic of the *ruber* group (Fig 3). Thus, although the shape of the primary aperture does not differentiate “*Globigerinoides*” at species level, it allows the recognition of two different groups. Thus, molecular phylogenies, the fossil record, morphometric data on the early representatives of the group, and differences in the ontogeny of modern representatives all point to an independent origin for the *trilobus* and *ruber* groups (Fig 5). In view of such overwhelming evidence, we feel compelled to formally revise and amend the genera *Globoturborotalita*, and “*Globigerinoides*” and to reclassify the species of the *trilobus* group. For reasons outlined below, we do this by establishing the new genus *Trilobatus*.

The only existing genus level taxa associated with the *trilobus* lineages other than “*Globigerinoides*” are *Orbulina*/*Praeorbulina* and *Globigerinoidesella*. Of these, *Orbulina* d’Orbigny, 1839 would have priority. However, this genus represents a morphologically highly distinct descendant and an extended concept of *Orbulina* encompassing species without the distinct embracing final chamber would pose difficulties in finding a suitable synapomorphy for such genus. In addition, such a solution would be against the current practice of using large changes in the *Bauplan* of a shell or in the shape of the chambers in planktonic foraminifera to designate genera. Similarly, *Globigerinoidesella* El-Naggar, 1971 was erected to separate forms with radially elongated digitate protuberances on chambers from other species of "*Globigerinoides*". Its type species *Globigerina fistulosa* Schubert, 1910 possesses the same characteristics and it is not, therefore, representative of other species belonging to the *trilobus* lineage. The same applies to the two further species presently attributed to *Globigerinoidesella*, *G*. *hystricosa* (Belford, 1962) and *G*. *bollii* (Loeblich and Tappan, 1982) by [52]. Like in the case of *Orbulina*, the presence of radially elongated chambers is commonly considered a genus-level character (see [53]). In addition, we observe that the holotype of *Globigerina fistulosa* Schubert, 1910 is not available, and this lacking contrasts with the recommendation of the ICZN for the attributes of type species. On the contrary, a neotype of “*G*.” *trilobus* has been recently established by [54] (See S1 Table). Considering the lack of available names, we thus feel compelled to establish a new genus name for the *trilobus* group, using “*Globigerinoides*” *trilobus* as the type species.

## Systematic Taxonomy

Order Foraminiferida d’Orbigny, 1826

Superfamily Globigerinacea Carpenter, Parker and Jones, 1862

Family Globigerinidae Carpenter, Parker, and Jones, 1862

Subfamily Globigerininae Carpenter, Parker and Jones, 1862

### Genus *Globoturborotalita* Hofker, 1976 amended

*Zeaglobigerina* Kennett and Srinivasan, 1983, p. 42

*Type species Globigerina rubescens* Hofker, 1956

#### Description

Test moderate to high trochospiral, globigeriniform and generally evolute, consisting of 2 ½ to 3 ½ whorls; chambers slightly embracing 3 to 5 in the last whorl. Test profile is compact to lobate, circular to slightly ovate, with rounded peripheral margin. Chambers are globular to ovate increasing gradually to rapidly in size as added. The primary aperture is umbilical and arched, sometimes resembling an inverted droplet, rarely a low arch slit-like and tending to the peripheral margin. In some species the primary aperture may possess a thin lip, or a thick rim. The umbilicus is wide open and deep or narrow. Sutures radial to slightly curved on both sides. No supplementary apertures are present on the spiral side. The wall is spinose with a cancellate texture of *ruber/sacculifer*- or *sacculifer*-type. Differences and similarities between the genera *Globoturborotalita*, *Globigerinoides*, and new genus *Trilobatus* are outlined in Table 2.

**Table 2:** Similarities and differences between the Genera *Globoturborotalita* (ancestor), *Globigerinoides*, the new genus *Trilobatus* and its descendant *Globigerinoidesella*.

	Genus *Globoturborotalita*	Genus *Globigerinoides*	Genus *Trilobatus*	Genus *Globigerinoidesella*
Coiling	Low to high trochospiral, evolute	Low to high trochospiral, evolute	Low trochospiral, intitially involute, later evolute	Low trochospiral, intitially involute, later evolute
Number of whorls	2½ to 3 ½	2½ to 3	3	3
Chambers in the last whorl	3 to 5	3 to 4	3; rarely 4	4
Last chamber	Normally symmetrical, globular to ovate	Globular to ovate symmetrical to radially compressed and asymmetrical	Globular to ovate symmetrical to enlarged and embracing or irregular and laterally compressed	Globular to ovate, irregular, laterally compressed, typically with one to numerous digitate extensions
Profile; periphery	Lobate; rounded	Lobate; rounded	Compact to lobate; rounded	Strongly lobate, characterized by numerous digitate extensions
Outline	Circular to slightly ovate	Subcircular to slightly ovate or subtriangular, to subrectangular	Ovate to subtriangular or subrectangular	Ovate to subtriangular
Sutures	Straight to slightly curved on both sides	Straight to slightly curved on both sides	Straight to slightly curved on both sides	Straight to slightly curved on both sides
Primary aperture	Umbilical, generally wide and arched, rarely tending to the peripheral margin, sometimes bordered by a rim	Umbilical, generally wide and arched, sometimes bordered by a thin lip	Umbilical-extraumbilical elongated slit, sometimes moderately high arched	Umbilical-extraumbilical elongated asymmetrical high arch, bordered by a lip
Supplementary apertures	Absent	Generally present and arched, placed at the intersection of the spiral and radial sutures, rarely absent, sometimes bordered by a thin lip. They may be one or two per chamber.	Slit-like or low to high arched, placed at the intersection of the spiral and radial sutures. Sometimes not visible, one per chamber.	Slit-like or low to high arched, placed at the intersection of the spiral and radial sutures. Sometimes not visible, one per chamber.
Umbilicus	Wide open and deep or narrow	Wide and open in most species	Narrow and concealed	Narrow and concealed
Wall texture	*ruber/sacculifer*- or *sacculifer*-type	*ruber-* or *ruber/sacculifer*-type	*sacculifer*-type	*sacculifer*-type

#### Remarks

Some representatives of this genus were previously attributed to *Zeaglobigerina* (Kennett and Srinivasan, 1983) and not *Globoturborotalita* (Hofker, 1977). However, the revision by [50] showed that the concept of the genus *Globoturborotalita* including information on wall texture can accommodate the *Zeaglobigerina* lineage of [7]. The genus first appears close to the Paleocene—Eocene thermal maximum event in the Eocene Zone E1 [50] and it is still present in the modern oceans. The current definition of the genus as amended by [50] includes the species: *Globoturborotalita bassriverensis* Olsson and Hemleben, 2006; *G*. *gnaucki* (Blow and Banner, 1962); *G*. *martini* (Blow and Banner, 1962); *G*. *brazieri* (Jenkins, 1966); *G*. *cancellata* (Pessagno, 1963); *G*. *connecta* (Jenkins, 1964); *G*. *euapertura* (Jenkins, 1960); *G*. *labiacrassata* (Jenkins, 1966); *G*. *woodi* (Jenkins, 1960); *G*. *druryi* (Akers, 1955), *G*. *nepenthes* (Todd, 1957); *G*. *decoraperta* (Takayanagi and Saito, 1962) and *G*. *rubescens* (Hofker, 1956).

#### Distinguishing features

Differences and similarities between the genus *Globoturborotalita*, *Globigerinoides*, *Globigerinoidesella*, and *Trilobatus* n. gen. are outlined in Table 2.

### Genus *Globigerinoides* Cushman 1927 amended

Type species *Globigerina rubra* d’Orbigny, 1839

#### Description

Test low to high trochospiral, globigeriniform and generally evolute, consisting of 2½ to 3 whorls. The peripheral margin is rounded, the test outline is from subcircular to slightly ovate or subtriangular to subrectangular and lobate. Chambers are generally globular to ovate, but may become radially compressed and asymmetrical, three to four in the last whorl increasing gradually in size as added. The primary aperture is umbilical and placed in a generally wide and open umbilical area; supplementary apertures are present on the spiral side, they may be one or more and are placed at the intersection of the spiral and radial sutures. Thin lips may be present on the primary and supplementary apertures. The wall texture is cancellate, irregular honeycomb, with spines irregularly distributed, it may be *ruber*-type or *sacculifer/ruber*-type sensu [22].

#### Distinguishing features

Differences and similarities between the genus *Globoturborotalita*, *Globigerinoides*, *Globigerinoidesella*, and *Trilobatus* n. gen. are outlined in Table 2.

#### Remarks

The genus was erected by [9] and described as similar to *Globigerina* but possessing numerous and large supplementary apertures on the spiral side of the last whorl only. Bolli [26] also informally included in the genus species with supplementary aperture on the spiral side in chambers from the inner whorls. Blow [55] emended the description of [26] and excluded from the genus *Globigerinoides* all the Paleocene species such as *Globoconusa daubjergensis* Brönnimann, Eocene species with the exception of *Globigerinoides higginsi* Bolli (now *Guembelitrioides nuttalli* (Hamilton)—see p. 84 in [50]) and all Oligocene species. He considered as *Globigerinoides* only Neogene species with several spiral supplementary apertures in chambers prior to the last with the exception of the phylogenetically primitive *Globigerinoides quadrilobatus primordius* Blow and Banner, which possesses only one. The genus first appears at the Oligocene-Miocene transition, and in particular at the base of Subzone M1a [13] and is still present in the modern oceans. Although some taxonomic revision may be needed, and the following list may not be comprehensive we include in the new definition of the genus the species: *Globigerinoides bollii* Blow, 1959; *G*. *italicus* Mosna and Vercesi, 1975; *G*. *obliquus* Bolli, 1975; *G*. *extremus* Bolli and Bermudez, 1965; *G*. *quadrilobatus* (d'Orbigny, 1846); *G*. *subquadratus* Brönniman and Resig, 1971; *G*. *ruber* (d'Orbigny, 1839); *G*. *elongatus* (d'Orbigny, 1839); *G*. *bulloideus* Crescenti, 1966.

### Genus *Trilobatus* Spezzaferri, Kucera, Pearson, Wade, Rappo, Poole, Morard and Stalder new genus

Type species *Globigerina triloba* Reuss, 1850

#### Description

Test low trochospiral, involute and compact in the initial whorls, later becoming slightly evolute to evolute. The test is from ovate to subtriangular or subrectangular to slightly lobate in outline with rounded peripheral margin with three to four subspherical chambers in the last whorl, increasing rapidly in size. The last chamber may become embracing and comprise one half of the test or it may become irregularly shaped and flattened. Sutures are depressed, straight to slightly curved on both sides, the umbilicus is often narrow and concealed. The primary aperture is an umbilical-extraumbilical elongated slit, tending toward the margin in many species. Supplementary apertures on the spiral side are irregular slits or low arches placed at the intersection of the spiral and radial sutures. The wall texture is cancellate, spinose and *sacculifer*–type sensu [22].

#### Distinguishing features

Differences and similarities between the genus *Globoturborotalita* Hofker, 1976, *Globigerinoides* Cushman, 1927, *Globigerinoidesella* El-Naggar, 1971 and new genus *Trilobatus* are outlined in Table 2. The descendant *Praeorbulina* differs from *Trilobatus* by possessing a last chamber tending to envelope the entire test and *Globigerinoidesella* by possessing irregular digitate projections on the final chambers. The potentially descendant lineage *Sphaeroidinellopsis*—*Sphaeroidinella* differs by developing a thick cortex covering the test surface.

#### Remarks

Hemleben et al. [56] demonstrated that specimens attributed to *T*. *trilobus* and *T*. *sacculifer* are one single biological species based on culturing evidence. Their observation was later proved by genetic evidence [24]. These authors have recommended for all modern representative of this genetic species the name *sacculifer* because it was the only one described within modern plankton [57]. They discuss also the possible priority of the name *G*. *quadrilobatus* as senior synonym. However, considering the possibility that the fossil representatives of the plexus with different morphologies could represent different species, they have proposed to retain *trilobus* for fossil populations and use the name *sacculifer* for all modern specimens of the plexus, with the use of ‘‘with sac” or ‘‘without sac” for the description of morphotypes within this species.

New observations of the PPFWG on *G*. *quadrilobatus* and its lectotype documented in [58] have revealed that this species is not related to the *trilobus/sacculifer* but rather to the *ruber* lineage and therefore, it cannot be a senior synonym of *trilobus/sacculifer*. The holotype of this species was never described by its author [59], therefore [15] designated a lectotype, which no longer exists. This specimen was attributed to the “*G*. *trilobus*” group (Blow and Banner 1962; [8, 58]). Papp and Schmid [58] designated a lectotype from the original material of [59]. In particular, new SEM images of this lectotype shows a highly arched and symmetrical aperture, centered in the umbilicus, a *ruber/sacculifer*-type wall texture with strong affinities to the *G*. *ruber* group.

The occurrence of specimens with a tendency to develop a sac-like final chamber can be traced to the early Miocene (Fig 5), but their occurrence throughout the fossil record is often inconsistent, rendering the recommendation to keep both *trilobus* and *sacculifer* unnecessary. In line with [24], the development of the sac-like final chamber is considered as a phenotype. However, in the fossil record it is useful to separate these forms, which have different ecological preferences, and thus, to simplify the use of the new genus *Trilobatus* we consider acceptable naming them *T*. *sacculifer* and *T*. *trilobus*.

The name *Trilobatus* for the new genus has been chosen because it recalls the name of the designated type species *T*. *trilobus*, aiming to reduce confusion when searching on names in taxonomic databases and the literature. The first representative of this genus is *T*. *primordius*. Its presence within *Paragloborotalia opima* (Zone O5) reported by [60] is not presently proven. Many other authors (see [12] for a comprehensive list of references) reported its lowest occurrence in the late Oligocene Zone O6-O7. Berggren et al. [61] place the first appearance datum (FAD) of this species at 26.7 Ma and it Last Common Occurrence (LCO) at 24.3 Ma (as per the magnetochronology of [30]). The genus diversifies at the base of Zone M1 (Fig 1) with the appearance of *T*. *trilobus* equated at 23.73 Ma [13]. The definition of the genus *Trilobatus* presently includes the species: *T*. *immaturus* (LeRoy, 1939); *T*. *praeimmaturus* (Brönnimann and Resig, 1961); *T*. *primordius* (Blow and Banner, 1962); *T*. *subsacculifer* (Cita, Premoli Silva and Rossi 1965); *T*. *trilobus* (Reuss, 1850); *T*. *bisphericus* (Todd, 1954); *T*. *sicanus* (de Stefani, 1952); *T*. *sacculifer* (Brady, 1877). Note: We have followed the attribution of [62] who do not include *T*. *sicanus* in the genus *Praeorbulina*. This species is here therefore attributed to *Trilobatus*.

## Summary

Fossil and genetic evidence on the phylogeny of Neogene globigeriniform planktonic foraminifera with supplementary apertures is employed to distinguish two main groups of taxa derived from *Globoturborotalita* in the late Oligocene and early Miocene that independently evolved supplementary apertures on the spiral side of the test. In consequence of this observation, we amend the genus description of *Globigerinoides* and erect *Trilobatus* as a distinct new genus to separate the *trilobus* and *ruber* clades.

## Appendix

For the classification of the wall textures we have followed the concept initiated by [63] and developed by the PPFWG since 1989 and summarized in [64] as follows:

*sacculifer*-type: wall textures with pores equally distributed, the wall is typically cancellate with strongly symmetrical honeycomb structure. Spines are placed at the intersection of ridges supported and surrounded by lamellar growth. As spines are reabsorbed during gametogenesis, spine holes are left behind. However, gametogenetic growth may obscure spine holes [22].

*ruber*-type: the cancellate structure of this wall is not as symmetrical as in the *sacculifer*-type, spines are also thinner and less regularly distributed.

*ruber/sacculifer*-type: this cancellate wall texture may be strongly symmetrical on some parts of the test and asymmetrical on the other parts.

## Supporting Information

S1 Table Summary of the investigated sites, with coordinates, water depth (when relevant), investigated core numbers (a minimum of three samples per section for each core have been investigated). All DSDP and ODP cores are stored at DSDP and ODP Repositories BCR = Bremen Core Repository, Germany; GCR = Gulf Coast Repository, USA; KCR = Kochi Core Repository, Japan). Samples are stored at the University of Milano Dipartimento Scienze della Terra “Ardito Desio, Via Mangiagalli 34, 20133 Milano, Italy (UniMi) at the University of Fribourg, Department of Geosciences, Chemin du Musée 6, 1700, Fribourg, Switzerland (UniFr) or at the University of Vienna, Department of Palaeontology, Althanstraße 14, 1090 *Vienna* (UniVie). In the table are also the details of the type specimen of the new genus *Trilobatus*.
(XLSX)

S2 Table Morphometric data on the group “*Globigerinoides*” from samples DSDP Hole 538-2CC. (XLSX)

S3 Table Morphometric data on the group “*Globigerinoides*” from samples DSDP Hole 151-5-2. (XLSX)

S4 Table Morphometric data on the group “*Globigerinoides*” from samples PJ262 (K3 F40-78). (XLSX)
