# Correction: Fossil and Genetic Evidence for the Polyphyletic Nature of the Planktonic Foraminifera "*Globigerinoides*", and Description of the New Genus *Trilobatus*

**DOI:** 10.1371/journal.pone.0259924

**Published:** 2021-11-09

**Authors:** Silvia Spezzaferri, Michal Kucera, Paul Nicholas Pearson, Bridget Susan Wade, Sacha Rappo, Christopher Robert Poole, Raphaël Morard, Claudio Stalder

In the Systemic Taxonomy section there is a subsection missing. The subsection should be titled Nomenclatural acts and should come after the Remarks subsection. The Nomenclatural acts subsection should read as follows: The electronic edition of this article conforms to the requirements of the amended International Code of Zoological Nomenclature, and hence the new name contained herein is available under that Code from the electronic edition of this article. This published work and the nomenclatural act it contains have been registered in ZooBank, the online registration system for the ICZN. The ZooBank LSIDs (Life Science Identifiers) can be resolved and the associated information viewed through any standard web browser by appending the LSID alphanumeric component to the prefix “http://zoobank.org/”. The LSID for this publication is: urn:lsid:zoobank.org:pub:B0ABE4D9-579F-4E40-B4B6-AB396B41F409. The electronic edition of this work was published in a journal with an ISSN, and has been archived and is available from the following digital repository: PubMed Central.

There is information missing from the section Genus *Trilobatus* Spezzaferri, Kucera, Pearson, Wade, Rappo, Poole, Morard and Stalder new genus. There should be a line immediately under this title that reads as follows: urn:lsid:zoobank.org:act:0D088398-B3AB-40CE-9011-0B8B7D977D2D
